# Moving beyond forest cover: Linking forest density, age, and fragmentation to diet

**DOI:** 10.1007/s12571-025-01535-7

**Published:** 2025-04-08

**Authors:** Aeryn Ng, Sarah E. Gergel, Maya Fromstein, Terry Sunderland, Hisham Zerriffi, Jedidah Nankaya

**Affiliations:** 1 Department of Forest and Conservation Sciences, Faculty of Forestry, The University of British Columbia, Vancouver, Canada; 2 Department of Forest Resources Management, Faculty of Forestry, The University of British Columbia, Vancouver, Canada; 3 School of Natural Resources, Environmental Studies and Agriculture, Maasai Mara University, Narok, Kenya

**Keywords:** Diet diversity, Food security, Forest fragmentation, Landscape ecology, Landcover, Remote sensing

## Abstract

Forests support food security and nutrition worldwide, especially so for highly forest-dependent communities who collect a variety of food products from nearby forests. While the importance of forest cover to the diets of forest-dependent communities has been well-researched, little is known regarding the role of more specific forest characteristics – information that would be valuable for better identifying the landscapes that support a nutritious and diverse diet. To address this research gap, we linked child dietary data to remotely-sensed geospatial indicators of surrounding forest characteristics – using more nuance than is typically undertaken – by examining forest age, tree density, and forest fragmentation in Kenya’s East African Montane Forests. Interestingly, dietary diversity of children demonstrated no or relatively weak associations with forest characteristics. However, by parsing out individual food groups, we exposed the nuance and complexities associated with the forest-diet relationship. Vegetable/fruit consumption was positively associated with open and moderately dense forest cover, but negatively associated with fragmented forest cover. The consumption of meat and vitamin A-rich fruit was positively associated with younger forest cover, and negatively associated with dense forest cover. Older forest cover was positively associated with green leafy vegetable consumption, but negatively associated with other vegetable/fruit consumption. Our findings provide suggestive evidence that there is no single ‘ideal’ type of forest for supporting food security and nutrition – rather, different types of forests are associated with different dietary benefits. Taken together, these results indicate the need for more in-depth research that accounts for factors beyond the proximity and amount of generic forest cover.

## Introduction

### Food security and nutrition

Food and nutrition insecurity is a global challenge, with food insecurity affecting 815 million people worldwide (FAO et al., 2017). According to the FAO, food and nutrition security “exists when all people, at all times, have physical and economic access to sufficient, safe and nutritious food to meet their dietary needs and food preferences for an active and healthy life” (FAO, 1996). Notably, this definition emphasizes not only the quantity of food consumed, but also the quality. Historical efforts in supporting food security and nutrition have often focused on the availability of calories; however, caloric intake alone is not a holistic indicator of an individual’s food security and nutritional status (Hwalla et al., 2016; Jimenez Rincon et al., 2022; Zizza et al., 2008), as micronutrient intake must also be considered. Two billion people worldwide struggle with micronutrient (such as vitamin A, iron, iodine) deficiencies – an issue referred to as ‘hidden hunger’ (Development Initiatives, 2018).

Forests directly support the food security and nutrition of highly forest-dependent communities by acting as sources of micronutrient-rich wild foods (Powell et al., 2013). Globally, it is estimated that at least 1.6 billion people (Chao, 2012; Fedele et al., 2021; Newton et al., 2020) are forest-dependent. The consumption of wild foods is linked to higher vitamin A intake (Hall et al., 2019), a greater consumption of meat (Jendresen & Rasmussen, 2022), a greater consumption of dark green leafy vegetables (Cheek et al., 2023), and a higher dietary diversity (Cheek et al., 2023; Powell et al., 2011), demonstrating the important role forests may play in attaining a nutritious diet. Moreover, forests can be an especially important source of nutritious foods for the rural poor, who may not have access to markets but are often situated close to forest cover and thus wild foods (Sunderlin et al., 2007). Indirectly, forests support food security and nutrition via the provision of woodfuel for cooking food and boiling water, employment/income, and agricultural benefits such as pollination (Gitz et al., 2021; HLPE, 2017). Despite the recognition of forests as a contributor to food security and nutrition, agricultural expansion has often been the go-to strategy for improving food security – often at the expense of forest cover (Cerri et al., 2018; Kumeh et al., 2022; Meyfroidt, 2018; Wollenberg et al., 2011). Thus, in order to support the food security and nutrition of highly forest-dependent communities worldwide, forest conservation and management must be further centred within food system discussions (Cerri et al., 2018; Kumeh et al., 2022).

While numerous studies recognize the importance of forests in supporting a nutritious diet, most previous research focuses on the overall *proximity* and *amount* of forest (Alencar et al., 2022; Baudron et al., 2019; Hall et al., 2019; Ickowitz et al., 2014), with less attention paid to other characteristics of the forests (Hall et al., 2022; Rasmussen et al., 2020; Renó et al., 2016). Forest tree density, fragmentation, and age are all factors that may be linked to diet both directly and indirectly. Broadly, changes to these aspects of forest structure could alter the pathways – direct, agroecological, energy, market (Gergel et al., 2020) – in which forests are connected to diet, ultimately altering food and nutrition security. For example, Mitchell et al. (2015) posit that a fragmented forest allows for greater interspersion of human-dominated landscape patches amongst forest patches, thus making forests closer to humans and more physically accessible for resource collection (including forest foods and woodfuel). This theory is supported by the findings of Hall et al. (2022), which identify an increase in the consumption of fruits, vegetables, and vitamin A with an increase in forest fragmentation over time. Rasmussen et al. (2020), however, find edge density to be negatively associated with the consumption of wild fruits. The number of forest patches and forest patch size, on the other hand, was found to be positively associated with fruit consumption (Rasmussen et al., 2020). Renó et al. (2016) find an increase in forest fragmentation over time to be linked to a decline in agricultural productivity, potentially due to a decline in pollinator activity – indirectly correlating forests to diet. This lack of consistency amongst findings indicates the complex nature of the relationship between forests and diet, while also suggesting the importance of location-specific forest management strategies to best support the food security and nutrition of forest-dependent communities.

While forested landscapes are integral to achieving global food security and nutrition, currently, little is known regarding the ways in which the location of forest patches within a particular landscape, their size, age, and density play a role in the provisioning of forest foods. Within this broad context, our research attempts to address a research gap present in current literature regarding forest-diet relationships, leveraging the opportunities provided by remote-sensing to track forest changes. This research maintains particular emphasis on children’s diets – as the most nutritionally vulnerable members of a community (Ickowitz et al., 2014), the food security and nutrition of children is important to prioritize. Maintaining sufficient nutrient intake plays a significant role in supporting children’s physical and cognitive development, as well as preventing morbidity and mortality (Arimond & Ruel, 2004; Arimond et al., 2010; Black et al., 2003; Kant et al., 1993; Kennedy et al., 2007; Ruel, 2003). This research investigates the diets of rural children in Kenya’s Rift Valley and explores potential associations with forest cover, tree density, forest fragmentation, and forest age in the East African Montane Forest ecoregion. We aim to answer the question: **How do children’s diets differ with surrounding forest cover, tree density, forest fragmentation, and forest age?**

Through the use of remotely-sensed imagery and survey data, we aim to evaluate the potential dietary benefits provided by forests of varying sizes, densities, degrees of fragmentation, and ages. We first calculated forest cover, fragmentation, and age using landcover classifications derived from remotely-sensed Landsat imagery. Child survey data from the Demographic and Health Surveys (DHS) program (consisting of various diet metrics) were then analyzed in relation to these forest metrics. Through this research, we aim to go beyond forest cover and proximity alone, and gain insight on the benefits and drawbacks of fragmented, younger, sparsely-populated, or smaller forest patches in regard to a diverse and nutritious diet.

## Methods

### Study site: East African Montane Forests, Rift Valley, Kenya

**Fig. 1:**
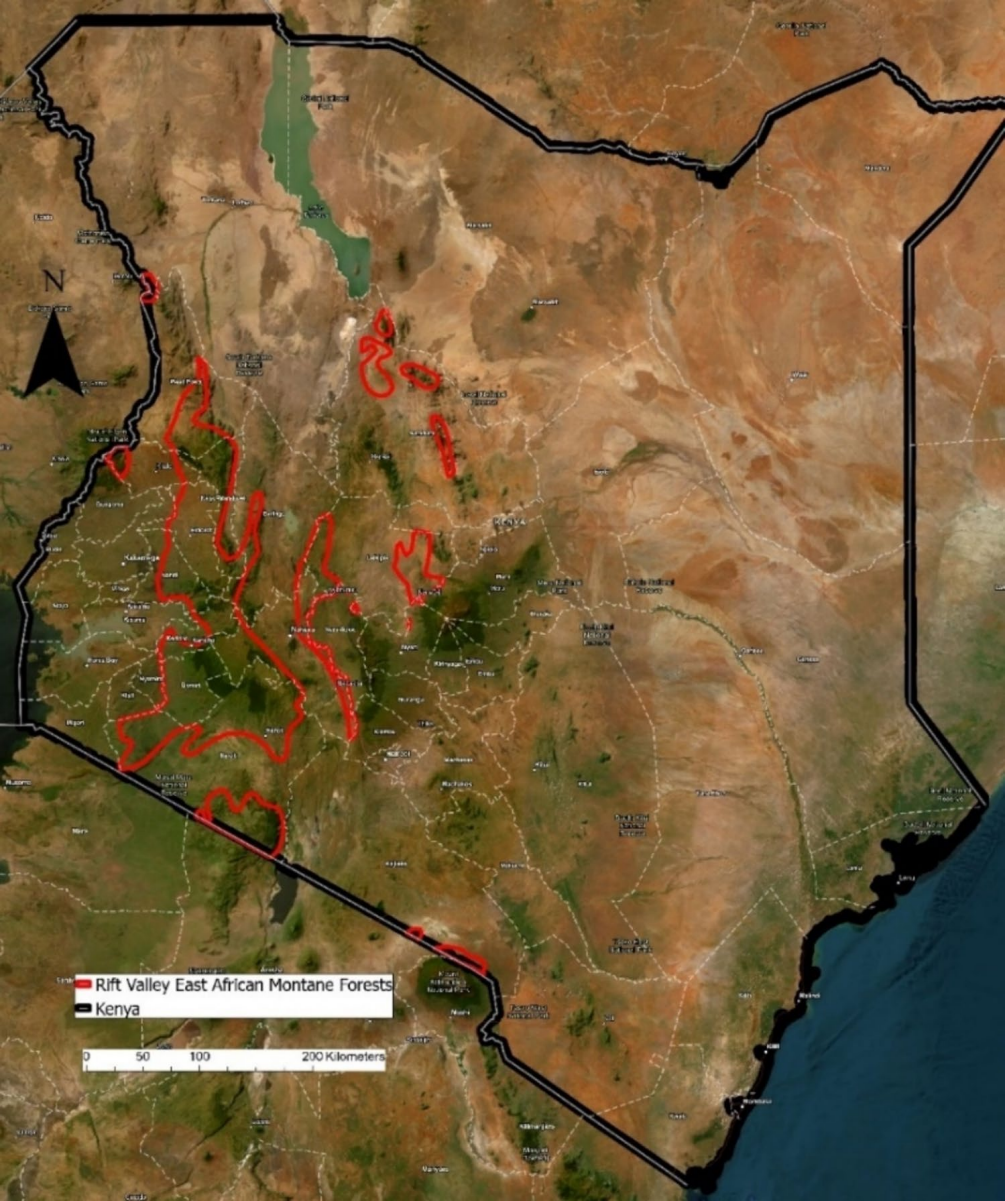
The study site: The East African montane forests of Kenya’s Rift Valley. Created using ArcGIS Pro (version 3.1.1)

The East African Montane Forests, an ecoregion dispersed across Sudan, Uganda, Tanzania, and Kenya, consists of forested areas at high elevations. These montane forests are home to numerous rural, forest-dependent communities facing food security and nutrition challenges. We look to investigate the diet of 338 children from rural households distributed throughout the East African Montane Forests of Kenya’s Rift Valley (Fig. 1), at elevations ranging from approximately 1400 m to 3300 m.

Globally, Sub-Saharan Africa has one of the highest rates of vitamin A deficiency (Mutie et al., 2020), an important vitamin for healthy immune systems in children, as well as for preventing night blindness and infectious illnesses. Vitamin A deficiency is one of the most common micronutrient deficiencies in Kenya, along with iron and zinc deficiencies. Iron deficiency impacts nearly 22% of Kenyan pregnant women, almost 10% of children aged 6–59 months old, and over 36% of children 5–14 years old (Government of Kenya, 2018). Roughly 70% of Kenya’s population is zinc deficient (Government of Kenya, 2018), which in children can impede growth and increase the risk of infection.

Precipitation in this region typically follows a bimodal pattern with longer, more intense rains from January to June, and shorter, less intense rains from November to December (Bartzke et al., 2018). The dry season occurs from July to October. Nonetheless, January and February are also sometimes referred to as the ‘short dry season’, as these months can also be dry (Bartzke et al., 2018). As a result of recent climate changes, precipitation patterns throughout Kenya are becoming increasingly variable and unpredictable (Gebrechorkos et al., 2020). Because precipitation is becoming less predictable, it is now difficult for local farmers and pastoralists to plan for and adapt to drought (Gebrechorkos et al., 2020). In such cases, forest foods can act as an increasingly important safety net during seasonal food shortages (Shackleton & Shackleton, 2004; Wunder et al., 2014). Multiple nutrient-dense edible plant species grow throughout the region’s forests; however, the main cultivated crop in this region is maize – a shade intolerant species requiring tree canopy removal for optimal growth (Capitani et al., 2019; Hamunyela et al., 2020). Agroforestry practices are also common throughout this region, with some of the most prevalent tree species being *Grevillea robusta, Cupressus lusitanica,* *J. procera*, *Croton megalocarpus*, *Callistemon citrinus*, *Schinus molle*, *Plectranthus barbatus*, and *Euclea divinorum* (Kehlenbeck et al., 2011).

Due to agricultural expansion, as well as human encroachment, logging, and political patronage, forests within this region have experienced significant degradation and fragmentation over time (Klopp & Sang, 2011; Miller et al., 2021). As a result, forests within this region currently consist of both old-growth and younger forests, as well as both larger, intact forest patches and smaller, more fragmented patches. Across Africa, projects such as The Great Green Wall and AFR100 are working to restore forested landscapes; however, little is known about the impacts that landscape restoration and reforestation activities (and thus changes to forest stand density, fragmentation, or age) may have on forest food production and accessibility – thus potentially posing a risk to the food security.

### Quantifying diet using demographic and health surveys data

We analyzed surveys collected by the Demographic and Health Surveys (DHS) program from 2014 for 338 rural households dispersed throughout the East African montane forests of Kenya’s Rift Valley. In particular, the unit of analysis was the oldest child in each household. We analyzed diet data of the oldest child in each household (rather than averaging across all children within a household) in order to focus on children old enough to consume forest foods and avoid children whose diets may consist primarily of breast milk (Mbagaya, 2009; Talbert et al., 2020). DHS collected this data by asking mothers to report whether each of her children had consumed foods from each food group (Table 1) within the past 24 h (reported as a binary response: ‘yes’ or ‘no’). The children included in this study ranged from 0 to 59 months old, with an average age of 29.9 months.

**Table 1:** Example of integration of FAO food groups (FAO, 2021; Hall et al., 2022) with concordant categories collected by child-level Demographic Health Survey (Kenya National Bureau of Statistics et al., 2015)

FAO food groups	DHS food groups
1) Grains, white roots, tubers, plantains	1.a) Grains 1.b) Roots and tubers
2) Pulses	2) Pulses and nuts
3) Nuts and seeds	
4) Dairy	3.a) Milk 3.b) Other dairy products
5) Meat, poultry, fish	4.a) Meat 4.b) Organ meat 4.c) Fish/shellfish
6) Eggs	5) Eggs
7) Dark green leafy vegetables	6) Dark green leafy vegetables
8) Other vitamin A-rich fruits and vegetables	7.a) Vitamin A-rich vegetables 7.b) Vitamin A-rich fruits
9) Other vegetables	8) Other vegetables and other fruits
10) Other fruits	

Firstly, we measured the dietary diversity of each child within the past 24 h. Consuming a wide variety of foods increases the likelihood of consuming an adequate amount of all essential nutrients. Thus, maintaining a diverse diet is an important aspect of nutrition. This is especially important for children, for whom a diverse diet has been linked to physical growth and cognitive development (Arimond & Ruel, 2004; Ruel, 2003). Dietary diversity, measured as the number of different food types consumed by an individual within a specified amount of time, is thus an important consideration with regards to dietary quality and nutrient intake (Ickowitz et al., 2014).

To measure dietary diversity, we used the food groups outlined by the Food and Agriculture Organization of the United Nations (FAO) (Table 1) (FAO, 2021). These guidelines represent the current standard for calculating women’s (aged 15 to 49 years) dietary diversity in low- and middle-income regions, and are routinely modified and implemented in both household and child dietary diversity research (Hall et al., 2022; Madzorera et al., 2020; Rasmussen et al., 2020). FAO guidelines for assessing infant and young child dietary diversity are intended for children aged 6 to 23 months, and implement the use of almost the same food groups as those for assessing women’s dietary diversity, with the principal exception being the inclusion of breastmilk (FAO, 2021). As our study population includes children aged up to 59 months (an age at which breastmilk might no longer be consumed), we have opted to use the food groups recommended for calculating women’s dietary diversity – however, it should be noted that this may result in an underestimation of children’s dietary diversity in the cases in which breastmilk is being consumed. Dietary diversity of the oldest child was quantified by assigning 1 point for every food group reported as consumed, with more points indicating a higher dietary diversity (as done by Hall et al., 2022; Rasmussen et al., 2020).

While the FAO recommends calculating dietary diversity using 10 food groups, DHS assesses 13 food groups for children. Furthermore, DHS food groups are not identical to those of the FAO. Therefore, we re-grouped DHS food groups to be concordant with appropriate FAO food groupings, resulting in the calculation of children’s dietary diversity using eight food groups (Table 1).

### Identifying spatial trends in forest cover, age, and fragmentation

We characterized geospatial forest characteristics within 10 km radii of households. We do not expect most individuals to travel more than 10 km to access food, making 10 km an appropriate radius distance (Hall et al., 2022; Layton et al., 1991). The forest characteristics we chose to focus on were those with potential relevance to child diet. Specifically, we looked at clusters of households and determined the area of contemporary forests surrounding each cluster, and further categorized forests according to stand density, degree of fragmentation, and age, as these characteristics have the potential to be associated with the diet of local forest-dependent communities (Berens et al., 2008; Höhl et al., 2020; Laestadius et al., 2015; Mitchell et al., 2015; Padilla et al., 2009; Renó et al., 2016; Romero-Duque et al., 2007).

To protect the anonymity of households, DHS creates clusters of households based on census enumeration areas (typically a village or group of villages in rural settings). Households within a cluster are all assigned the same location using the centroid of the enumeration area (Burgert et al., 2013). For reporting purposes, the locations of 99% of cluster centroids are offset by up to 5 km (0–5 km), while 1% are randomly selected for offsetting by as much as 10 km (0–10 km). As a result, the 338 households surveyed in this dataset were grouped by DHS into 126 clusters (comprising 1 to 7 households per cluster). All geospatial determinations (for forest cover and roads, etc.) were determined at the cluster (not household) level. Although the clustering of households creates a source of uncertainty in household locations (as intended), the results of the geospatial forest measurements were likely not greatly impacted because the 10 km radii built around each cluster still likely captured many of the landscapes surrounding the original households as well as the cluster locations, most of which were only offset by < 5km.

To calculate forest characteristics, we used Landsat-derived land cover classifications (30 m resolution) from the Kenya Department of Resource Surveys and Remote Sensing who implemented a minimum mapping unit of 0.5 hectares (i.e., 5000 m^2^). This classification distinguishes three distinct forest density categories: “dense” (canopy cover over 65% of forest), “moderate” (40% < 65%), and “open” (15% < 40%), and is available for our study site for 1990, 2000, 2010, and 2014.

**Fig. 2:**
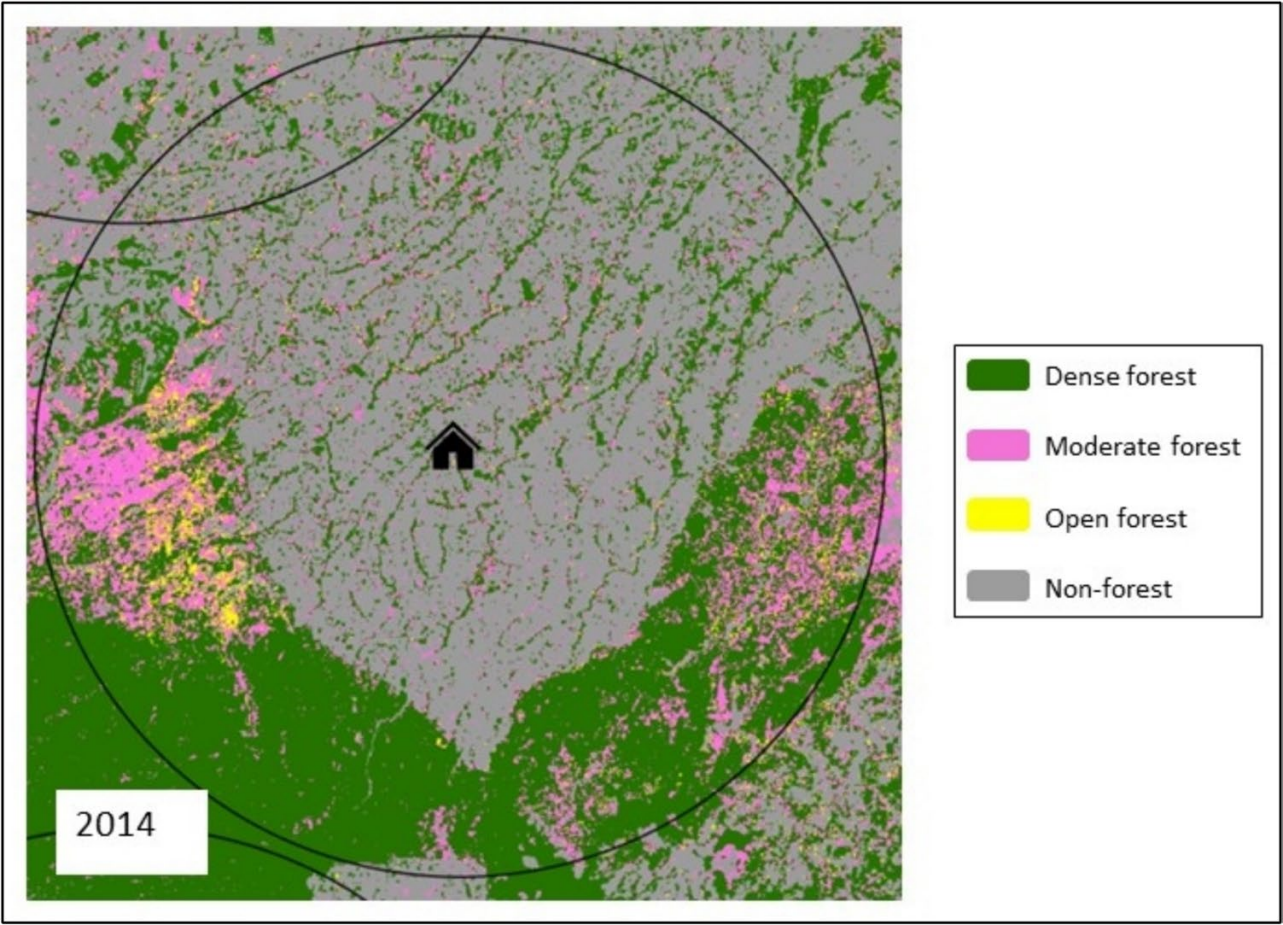
An example of a household cluster, 10 km radius, and surrounding forest cover (dense, moderate, open) in 2014. Created using ArcGIS Pro (version 3.1.1)

We first determined the proportional cover occupied by each of the canopy cover density classes within a 10 km radius of each household cluster in 2014 (the same year as DHS survey collection) (Fig. 2) using ArcGIS Pro (version 3.1.1). We used the same canopy cover density classes as those used by the Kenya Department of Resource Surveys and Remote Sensing, as well as the Intergovernmental Panel on Climate Change guidelines on landcover change inventory (Eggleston et al., 2006). As mentioned above, a 10 km radius was used for two reasons. First, this distance helps account for the potential 10 km location offset of household clusters (Hall et al., 2022). Furthermore, we do not expect most individuals to travel more than 10 km to access food, making 10 km an appropriate radius distance (Hall et al., 2022; Layton et al., 1991).

Secondly, using the 2014 Landsat-derived land cover classifications, we quantified forest fragmentation within a 10 km radius of each household cluster using the ‘landscapemetrics’ package (Hesselbarth et al., 2019) in R 4.3.1 (R Core Team, 2023). Forest patches were defined as a minimum of 15% tree canopy cover (Regional Centre for Mapping of Resources for Development, 2016). Forest fragmentation was quantified as the perimeter to area ratio of each forest patch, with a higher value indicating greater fragmentation. The mean perimeter to area ratio was then determined for each 10 km radius and assigned to the corresponding household cluster.

Lastly, we performed change detection analyses with the annually available landcover data from 1990, 2000, 2010, and 2014 in order to determine the age (measured via the first appearance) of each forest patch. For each forest patch in the 2014 image, we used the historical images to determine when each forest patch was established (to the nearest decade). Each forest patch was then classified into age ranges from: 0 < 4; 4 ≤ 14; 14 ≤ 24; or > 24 years (these age ranges were determined based on the availability of landcover data). Finally, we determined the proportional coverage of these different age classes in 2014 within a 10 km radius of each household cluster.

### Covariates

In addition to forest attributes, we also examined several co-variates. First, we determined the Euclidean distance between each household cluster and the nearest major road (paved or unpaved) as represented from the Digital Chart of the World (Defense Mapping Agency, 1993). Shorter distance to roads, and thus markets, may be associated with a higher dietary diversity if households have access to additional nutritious foods they would not otherwise consume (Luckett et al., 2015; Shively, 2017) but may increase the consumption of processed foods and thus not improve micronutrient consumption (Reyes-García et al., 2019). The month in which the survey took place was classified as either the wet or dry season and included in the diet models as a dummy variable. Seasonality is an important consideration when modelling diet in order to account for the seasonality of food production, as certain species of fruits or vegetables may only be produced in the rainy season. In addition, DHS data includes the age of each surveyed child, the number of members in each household, and the month in which the survey took place, all of which we accounted for when modelling diet-forest relationships. The number of household members was considered, as a higher number of household members could alter the availability of food for each individual member (Gitagia et al., 2019; Korir et al., 2023).

Finally, DHS data also provides an index of household wealth – a metric commonly associated with diets. Ickowitz et al. (2014) found that children from wealthier households were more likely to have a greater dietary diversity, and consume more fruits, vegetables, and animal-sourced foods. Household dietary diversity has also been found to be positively related to household wealth (Korir et al., 2023); however, preadolescents from wealthier families have demonstrated an increased consumption of refined foods and sugar relative to those from less wealthy families (Korir et al., 2023). In contrast, the preadolescents’ consumption of healthier food groups (i.e. beans, nuts, dairy, roots and tubers, fish and seafood, wholegrains, fruits) is also associated with higher wealth (Kanerva et al., 2023). These patterns indicate the potential dietary benefits, as well as challenges, associated with wealth. To add further nuance, the importance of the role of wealth in diet is limited by market access. Additionally, while wealth has shown to be an important factor, rural, poor households may demonstrate a relatively high dietary diversity as they are often situated closer to forest cover and thus have more access to wild foods (Sunderlin et al., 2007). Note that the wealth scores assigned by DHS are calculated and standardized for each household at the national level rather than at the level of our study site. Relative to the entire country, more households in our study site are situated in the lower income categories.

### Statistical analyses: Diet-forests relationships

We identified the explanatory variables most strongly associated with 10 dietary response variables (Table 2) using general linear models based on N = 338 children, each with unique dietary information. However, because geo-locational information was only distinguishable at the cluster level (126 clusters), forest/land cover attributes were identical for all households within a cluster. Final models were chosen using backwards and forwards stepwise comparisons to identify the model with the lowest AIC value. All statistical analyses were completed using the base R package (stats version 4.3.1) (R Core Team, 2023) in R 4.3.1 (R Core Team, 2023) unless otherwise noted.

**Table 2:** Response and potential explanatory variables, data type, and model type

Diet variable category	Response variable	Data/model type	Potential explanatory variables
Dietary diversity in the past 24 h	Child dietary diversity (out of 8)	Counts (0 to 8) modelled via negative binomial regression	*Spatial variables:* • Proportion of dense/moderate/open forest within 10 km of household cluster • Mean perimeter to area ratio of forest patches within 10 km of household cluster • Proportion of forests in each age class (0 < 4; 4 ≤ 14; 14 ≤ 24; > 24 years) within 10 km of household cluster • Distance to nearest road *Non-spatial variables:* • Household wealth • Age of child • Number of household members • Season of interview (rainy vs. dry)
Child diet: Whether a specified food group was consumed in the preceding 24 h	Consumption of green leafy vegetables	Binary (yes/no) modelled via logistic regression	
	Consumption of vitamin A-rich fruits		
	Consumption of other fruits/vegetables		
	Consumption of meat		

Child dietary diversity data were modelled using negative binomial regressions using the ‘MASS’ package (Venables & Ripley, 2002) due to their count distributions (and to account for the under/overdispersion observed in the data). Due to their binary nature, we modelled the child diet data by fitting a generalized linear model with a binomial distribution and a logit link function.

Explanatory variables (Table 2) were scaled using the ‘dplyer’ package (Wickham et al., 2023) and collinearity of final models was evaluated using Kendall’s correlation coefficients. A high correlation between dense forest cover and forest > 24 years in age (0.7210) meant that we did not use these two explanatory variables in the same models. Furthermore, we did not include the total forest cover within a 10 km radius as an explanatory variable because it was highly correlated with both dense forest cover (0.7836) and forest cover aged > 24 years (0.7199). The ‘ggplot2’ package (Wickham, 2016) and ‘patchwork’ package (Pedersen, 2023) were implemented for data visualization purposes.

Finally, in order to address the clustering of households in the DHS data (and the resulting lack of independence between data points within a cluster), we calculated cluster-robust standard errors for coefficients of all final models (clustered at the DHS cluster level) using the ‘lmtest’ (Zeileis & Hothorn, 2002) and ‘sandwich’ packages (Zeileis, 2006; Zeileis et al., 2020). Confidence intervals and p-values of all coefficients were adjusted based on the value of the cluster-robust standard errors. Final models with cluster-robust standard errors were visualized using the ‘modelsummary’ package (Arel-Bundock, 2022).

## Results

### Household characteristics

This study included children residing in 338 different households, grouped into 126 clusters. Clusters consisted of a minimum of 1 to a maximum of 7 households. The mean number of households per cluster was 2.68, and the median number of households per cluster was 2.Just over half (54%) of clusters consisted of ≤ 2 households, and 73% of clusters consisted of ≤ 3 households.

The majority of households fell within the lowest (~ 33%) and second lowest (~ 35%) wealth quintile. Approximately 18% of households fall within the 3rd wealth quintile, approximately 12% of households fall within the 4th wealth quintile, and just over 2% of households fall within the highest wealth quintile.

The majority (~ 74%) of surveys took place in the dry season and conversely, just over a quarter of surveys took place in the wet season.

### Landcover characteristics

We measured three forest metrics within a 10 km radius of each household cluster: 1) the proportion of forest cover in each density class (open, moderate, dense); 2) the proportion of forest cover in each age class (0 < 4, 4 ≤ 14, 14 ≤ 24, > 24 years); and 3) the mean forest fragmentation (perimeter/area). Looking to the total forest cover across all 10 km radii of household clusters, the majority of forest cover fell into the ‘dense forest’ category (78%), whereas 12% fell into the ‘open forest’ category and 10% ‘moderate forest’ cover. Within a single 10 km radius, the minimum ‘dense forest’ cover was 0.1%, while the maximum was 45%. Moderate forest cover ranged from 0 to 15%. Open forest cover ranged from 0 to 25%. In terms of forest age, the majority of forest cover across all 10 km cluster radii fell into the ‘ > 24 years old’ category (46%), followed by ‘0 < 4 years old’ (31%), ‘4 ≤ 14 years old’ (13%), and ‘14 ≤ 24 years old’ (9%) category. Within a single 10 km radius, forest cover > 24 years old ranged from 0 to 38%. Forest cover aged 14 ≤ 24 years ranged from 0 to 10%. Forest cover aged 4 ≤ 14 years ranged from 0 to 7%. Forest cover aged 0 < 4 years ranged from 0.1% to 17%.

### Child diet: Forest is linked to vitamin A-rich fruit and meat consumption

The majority of children’s diets included meat but lacked fruits and vegetables. Approximately 43% of children consumed dark green leafy vegetables in the 24 h preceding the survey. In contrast, only 9% consumed vitamin A-rich fruits. Approximately 23% of children consumed other vegetables or fruits, and 92% of children consumed meat. Finally, the majority of children (approximately 86%) reported a dietary diversity of 4 out of 8 or less within the past 24 h. The FAO suggests a minimum dietary diversity score of 5 out of 8 to be used as a proxy for adequate micronutrient intake (FAO, 2021).

**Fig. 3:**
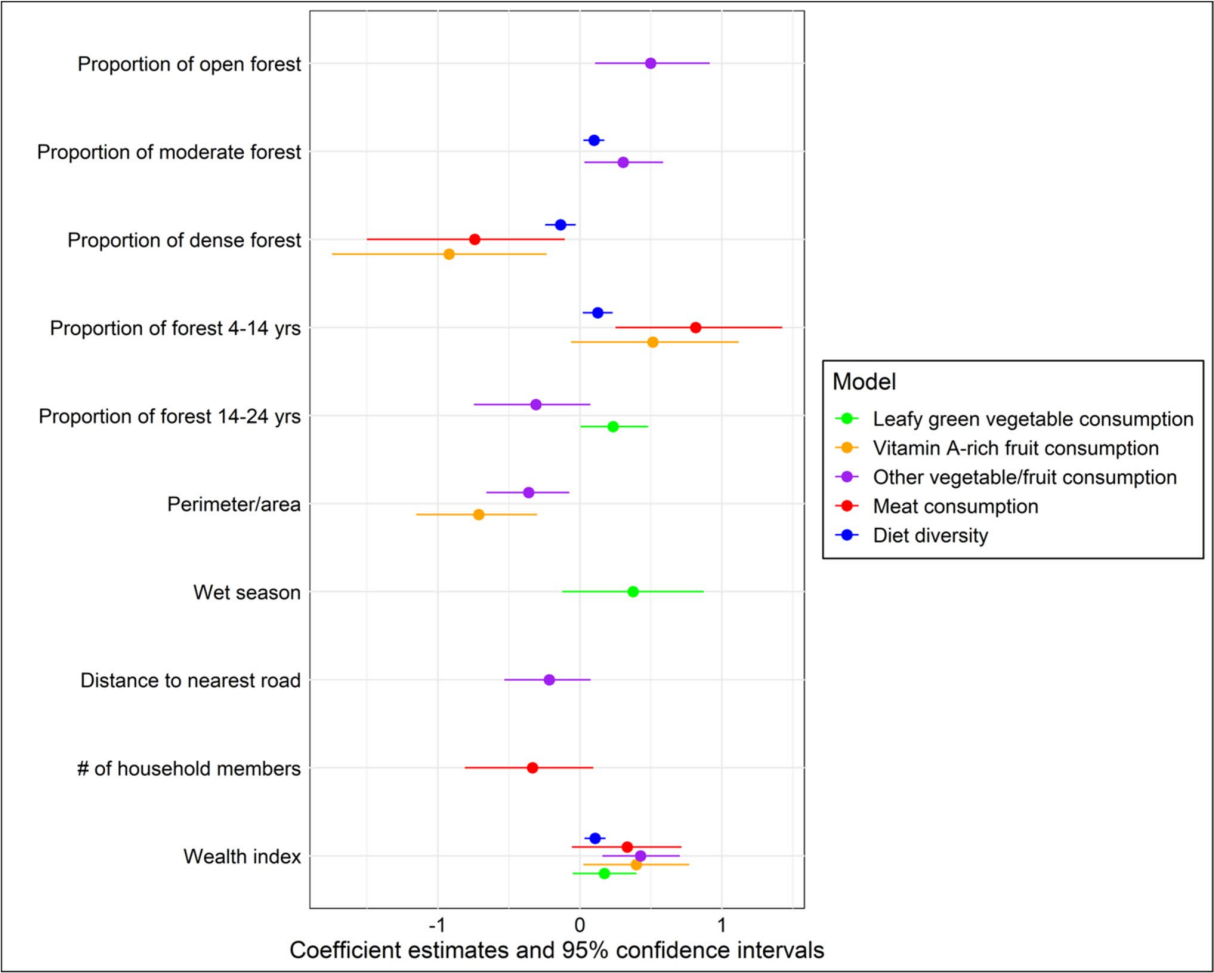
Explanatory variables included in the final model for each child diet response variable. Coefficients for each explanatory variable and cluster-robust standard errors indicated on the x-axis. (Note: coefficients from logistic regressions represent the change in log-odds, while coefficients from negative binomial regressions indicate the change in the log rate of occurrences. Both are on log scales.)

Based on the variable coefficients in the final logistic regressions (Fig. 3), children’s vitamin A-rich fruit consumption was the food group most highly associated with any single forest metric. In particular, children’s vitamin A-rich fruit consumption was most strongly negatively associated with dense forest cover, with a coefficient of −0.92. Vitamin A-rich fruit consumption was also negatively associated with forest fragmentation (with a coefficient of −0.71) and positively associated with forest cover aged 4–14 years (with a coefficient of 0.51). Children’s meat consumption exhibited similar patterns to vitamin A-rich fruit consumption with a positive association with forest cover aged 4–14 years (with a coefficient of 0.82), and a negative association with dense forest cover (with a coefficient of −0.74). Compared to these food groups, the consumption of other vegetables/fruits demonstrated relationships of relatively moderate strength with numerous forest metrics – in particular, a positive relationship with moderate (with a coefficient of 0.30) and open forest cover (with a coefficient of 0.50), and a negative relationship with forest fragmentation (−0.36). Children’s dark green leafy vegetable consumption was positively associated with forest cover aged 14–24 years (with a coefficient of 0.23), but relatively weakly so. Finally, while child dietary diversity was negatively associated with dense forest cover, and positively associated with moderate forest cover and forest cover aged 4–14 years, these relationships were relatively weak (with coefficients of −0.14, 0.10, and 0.13 respectively).

Household wealth index score was included in the final models for all child dietary response variables (Table 3, Fig. 3). Season was only included in the final model for the consumption of dark green leafy vegetables, but not found to be statistically significant (p-value > 0.1). Distance to nearest road was included in the final model for the consumption of other vegetables/fruits, but this relationship was also not deemed statistically significant (p-value > 0.1).

**Table 3:** Child diet response variables, explanatory variables included in the final models, and corresponding p-values

Response variable (diet within the past 24 h)	Explanatory variable	Direction of relationship (positive/negative)	P-value
Consumption of dark green leafy vegetables	Forest cover aged 14–24 years	+	0.0072
	Season (wet)	+	0.16
	Household wealth index score	+	0.16
Consumption of vitamin A-rich fruit	Dense forest cover	-	0.054
	Forest cover aged 4–14 years	+	0.081
	Mean forest fragmentation	-	0.0017
	Household wealth index score	+	0.12
Consumption of other vegetables/fruits	Open forest cover	+	0.012
	Moderate forest cover	+	0.0030
	Forest cover aged 14–24 years	-	0.15
	Mean forest fragmentation	-	0.025
	Distance to nearest road	-	0.24
	Household wealth index score	+	0.0028
Consumption of meat	Dense forest cover	-	0.097
	Forest cover aged 4–14 years	+	0.017
	Number of household members	-	0.098
	Household wealth index score	+	0.13
Dietary diversity (out of 8)	Moderate forest cover	+	6.763e-06
	Dense forest cover	-	0.024
	Forest cover aged 4–14 years	+	0.019
	Household wealth index score	+	0.014

## Discussion

Forests are an integral aspect of global food security and nutrition (Gitz et al., 2021; HLPE, 2017). Across a variety of countries, households’, women’s, or children’s consumption of wild foods is linked to a greater vitamin A intake (Hall et al., 2019), a greater consumption of meat and dark green leafy vegetables (Cheek et al., 2023; Jendresen & Rasmussen, 2022), and a higher dietary diversity (Powell et al., 2011). For children, a positive relationship between nearby forest cover and dietary diversity has been observed across twenty-one African countries (Ickowitz et al., 2014). Moreover, vitamin A consumption, as well as fruit and vegetable consumption has also been found to be higher in areas with greater forest cover (Hall et al., 2019, 2022). Despite the plethora of research in support of a positive association between forest cover and diet, this relationship is complex and nuanced. Furthermore, limited research investigates the forest-diet relationship while accounting for forest characteristics beyond cover. This information is critical in ensuring that forests are managed in ways that best support a nutritious and diverse diet for highly forest-dependent communities.

By leveraging remote-sensing and household survey data, this research adds to current literature by investigating more specific forest characteristics and their associations with the diet of local forest-dependent communities, asking: *How do children’s diets differ with surrounding forest cover, tree density, forest fragmentation, and forest age?* We investigated this question across the montane forests of Kenya’s East African Rift Valley. Overall, our findings indicate a complex relationship between forests and diet, with each forest characteristic demonstrating a different relationship with various aspects of child diet. The results of this study provide further insight on the nuances of the forest-diet relationship, a topic that is currently understudied.

### A complex relationship between dietary diversity and forests

A positive relationship between nearby forest cover and children’s dietary diversity has been identified in numerous countries (Galway et al., 2018; Ickowitz et al., 2014) – however, the analysis of forest density, fragmentation, or age is not commonly incorporated in this type of research. We found that the cover of dense forests and younger forests (aged 4 to 14 years) was positively associated with children’s dietary diversity, while the cover of moderate forests was negatively associated with children’s dietary diversity. However, these relationships were weak relative to the relationships we detected between forest characteristics and children’s consumption of fruit, vegetables, and meat. The magnitude of these relationships suggests that forest cover, density, and age may not always be strongly associated with the value of dietary diversity, despite previous literature identifying the importance of forest cover (Galway et al., 2018; Ickowitz et al., 2014). However, our findings may also differ from those of previous literature due to differences in methods as well as scale. Ickowitz et al. (2014), for example, examined forest-diet patterns via a multi-country analysis, using MODIS spatial data (with a resolution of 250 m). Regardless, our findings further emphasize the complex nature of the relationships between forests and diet, as well as the need for more in-depth research on this topic. The following sections further explore each forest characteristic and their relationships with individual food groups.

### Forest density is negatively associated with vitamin A-rich fruit consumption

Forest cover aged 14 to 24 years was positively associated with children’s consumption of green leafy vegetables, but negatively associated with the consumption of other vegetables/fruit. Forest cover aged 4 to 14 years was positively associated with children’s consumption of vitamin A-rich fruits, while dense forest cover was negatively associated with vitamin A-rich fruit consumption. Based on these results, forests of different ages may all be beneficial in supporting the consumption of different fruits or vegetables; however, it is interesting to note that dense forest cover (a variable highly correlated with total forest cover) was negatively associated with children’s consumption of vitamin A-rich fruits, demonstrating contrasting results to numerous previous studies that have found a positive relationship between total forest cover and vitamin A-rich fruit consumption (Hall et al., 2019, 2022; Ickowitz et al., 2014). This is indicative of the highly complex processes behind the forest-diet relationship. While total forest cover may frequently be positively associated with vitamin A-rich fruit consumption, the consideration of canopy density may introduce a new dimension that is lacking in current research. Overall, these findings provide suggestive evidence that not all forests are ‘equal’ with regards to supporting the diet and nutrition of local, forest-dependent communities.

Seasonality may play an important role in our findings, as the majority of the DHS surveys we analyzed were conducted in the dry season. This may have resulted in the loss of information on the consumption of certain wild foods that are only produced in the wet season, in particular fruit. However, when including seasonality as a variable in diet models, wet season was found to be positively associated only with the consumption of green leafy vegetables (with an insignificant p-value > 0.1).

### Forest fragmentation is negatively associated with fruit and vegetable consumption

We found forest fragmentation to be negatively associated with children’s consumption of vitamin A-rich fruits and other vegetables/fruits. These results partially align with the findings of Rasmussen et al. (2020), who identified a negative relationship between edge density and the consumption of wild fruits, but a positive relationship between number of forest patches and the consumption of wild fruits. While Mitchell et al. (2015) suggest that forest fragmentation could improve the physical accessibility of forest foods and thus increase their consumption, they also suggest that the supply of forest products may be reduced. Forest fragmentation has been found to be negatively associated with the supply of forest foods in the lower Amazon floodplain, specifically the supply of bushmeat species that prefer larger habitat patches (Renó et al., 2016). It is suggested that this decline in mammal species then led to the loss of important seed dispersers, and consequently the abundance of certain edible plant species as well (Renó et al., 2016). Thus, while it is possible that an increase in forest fragmentation may increase the physical accessibility of forest foods, the supply of forest foods being produced could have been negatively impacted, resulting in a decline in fruit and vegetable consumption. In order to gain a more in-depth understanding of the role of fragmentation, future research should incorporate the use of additional fragmentation metrics, as patch size and number of forest patches have been found to be positively associated with fruit and vegetable consumption, as well as with vitamin A intake (Hall et al., 2022; Rasmussen et al., 2020).

### Forest canopy density is negatively associated with meat consumption

Children’s meat consumption demonstrated a negative relationship with dense forest cover. In the case of meat consumption, an important source in Kenya comes from livestock (Mohajan, 2014). However, it is potentially more difficult to graze livestock in a denser forest, as more densely distributed trees may be less physically accessible for livestock to move through (Naveh & Kutiel, 1990; Riginos et al., 2012; Seligman & Perevolotsky, 1994). Duriaux Chavarría et al. (2018) found a positive relationship between a household’s forest proximity and the number of livestock they were able to support in Southern Ethiopia. They suggest that households closer to forests are more likely to supplement their livestock feed with forest biomass, thus allowing for larger herds (Baudron et al., 2017; Duriaux Chavarría et al., 2018); however, forest density was not investigated. Furthermore, a dense tree canopy may reduce the productivity of the grasses below, and thus the availability of forage for livestock (Riginos et al., 2012). Alternatively, a denser forest cover could be associated with a lack of livestock in the area (and thus a lower meat consumption), as livestock grazing in forests is related to a reduction in the tree density of the forest (Dufour-Dror, 2007; Piana & Marsden, 2014). The implications of forest density for nearby smallholder farms require more in-depth, place-based research.

In addition to livestock as a source of local meat consumption, bushmeat is another option. The consumption of bushmeat is a common practice across rural Africa (Davies, 2002). Bushmeat species commonly hunted for consumption across the study site (and neighboring regions) include species of wild shrews and rodents (Menz, 2014; Patel et al., 2023), and ungulates such as buffalo, impala, wildebeest (Gakuya et al., 2022; Mwakatobe et al., 2012). Previous literature indicates that bushmeat hunting may be associated with a reduction in tree density (Effiom et al., 2013; Jansen et al., 2010), as certain bushmeat species act as important seed dispersers of tree species in a forest ecosystem. It is suggested that as these bushmeat species are hunted, tree seeds are dispersed less, potentially leading to a reduction in tree density (Effiom et al., 2013; Jansen et al., 2010). Thus, dense forest cover could be negatively associated with bushmeat consumption. This provides another potential explanation to the negative relationship between meat consumption and dense forest cover we observed.

### Additional limitations & future research

While we have presented correlational evidence linking forest characteristics with child and household diets, the datasets we used were not sufficient for identifying the specific mechanisms underlying these relationships. A key limitation of the DHS dataset is the lack of differentiation of the source of consumed foods – that is, either the consumption of wild foods versus agriculturally-produced foods. Furthermore, DHS data does not differentiate between food that was foraged/cultivated by the survey respondent themself, or purchased. In addition, the land cover data we used to calculate the cluster-based forest metrics did not differentiate tree cover associated different land uses. Thus, any tree cover meeting the minimum canopy density threshold (15%) was classified as ‘forest,’ including natural forests, planted forests, agroforestry systems, and homegardens, As a result of the DHS and forest cover data limitations, we were unable to determine if tree cover surrounding households was likely cultivated/planted species, or whether such forests were mostly associated with consumption of wild foods, cultivated foods, purchased foods, or a combination of options. Therefore, mechanisms behind the relationships identified in this research require further investigation.

Secondly, the DHS diet surveys were primarily conducted in the dry season. This is important, as this could have resulted in the loss of information on the consumption of seasonal fruits and vegetables that are only produced in the wet season. We accounted for this by including seasonality as an explanatory variable in the dietary response variable models. Findings show children’s consumption of green leafy vegetables (Fig. 3) to be positively associated with the wet season (although not significantly). These findings suggest the possible importance of the wet season for the productivity of green leafy vegetable species (either wild or cultivated), and thus for their consumption. Future diet data collection should be conducted in both the dry and wet season in order to account for this important factor and further explore its influence.

A third limitation of this study was the inflexibility of the forest age data, which was restricted to available geospatial data from the years 1990, 2000, 2010, and 2014. Thus, for our study, the forest age classification scheme we used (0 < 4; 4 ≤ 14; 14 ≤ 24; or > 24 years) was chosen based solely on data availability and not on biophysical justifications. Nonetheless, we conducted additional sensitivity analyses to evaluate whether our model results would change with the use of different forest age classification schemes. To do so, we contrasted our original four forest age classes (0 < 4; 4 ≤ 14; 14 ≤ 24; or > 24 years) with model results using different age classification schemes of 0 ≤ 14; > 14 years and 0 ≤ 24; > 24 years. In doing so, we determined that models based on the new age classifications no longer included forest age variables as significant explanatory variables for meat and green leafy vegetable consumption. Moreover, for the consumption of other vegetables and fruits, the model using our original age classification scheme and the model using the classification scheme of 0 ≤ 24; > 24 years both identified the same explanatory variables as significant, whereas the model using the classification scheme of 0 ≤ 14; > 14 years identified different significant explanatory variables. For all other diet variables, models using the new forest age classifications were similar to our original models, with the inclusion of some different explanatory variables. Notably, forest fragmentation was not a significant explanatory variable in our original model of diet diversity; however, when evaluating the new forest age classification schemes, fragmentation was a significant explanatory variable in new diet diversity models and, therefore, may be an important factor to consider in future studies.

Overall, the primary contributions of this research are a suite of proposed hypotheses. Further research is required in order to rigorously test these hypotheses and establish causal relationships between the examined forest characteristics and dietary outcomes. Subsequently, research is needed to investigate the mechanisms behind our hypotheses. Future research should explore the sources of consumed foods (e.g., wild, agricultural), seasonal patterns, as well as additional points in time.

### Conclusion

This research highlights the complex relationships that exist between forests and diet. In concordance with previous literature (Baudron et al., 2019; Powell et al., 2013; Rasmussen et al., 2020), our findings provide suggestive evidence that forests support local food security and nutrition via a variety of different pathways, and that consideration of forest attributes beyond solely forest cover must be accounted for when considering the nutritional aspects of landscapes. By examining forest canopy density, forest age, and forest fragmentation, this research provides guidance on the near-term and long-term dynamics of the delivery of forest benefits – information that is valuable in identifying realistic scenarios in which forest restoration would be beneficial to forest-dependent communities.

Despite the valuable and irreplaceable benefits that forests provide, continued deforestation and forest degradation has resulted in a significant loss of forests globally. According to the FAO (2015), 129 million ha of forest have been lost worldwide between 1990 and 2015. Critical insight gained through community consultations (Höhl et al., 2020), as well as through the findings of this study, can be used to improve restoration plans whereby not only ecological functions are supported, but also the effective and long-term provisioning of food and nutrition.

Overall, this study contributes to the limited but growing literature on the important relationships between diet and forest density, age, and fragmentation. Our findings provide suggestive evidence that there is no single ‘ideal’ type of forest for supporting food security and nutrition – rather, different types of forests are associated with different dietary benefits. The results of this study will provide decision-makers with a better understanding of the potential for forest restoration to support forest-dependent diets, as well as recognize the value of types of forested landscapes for diet.

## Data Availability

The data that support the findings of this study are available from the corresponding author upon reasonable request.
